# Emergence of Members of TRAF and DUB of Ubiquitin Proteasome System in the Regulation of Hypertrophic Cardiomyopathy

**DOI:** 10.3389/fgene.2018.00336

**Published:** 2018-08-21

**Authors:** Ishita Gupta, Nishant K. Varshney, Sameena Khan

**Affiliations:** ^1^Structural Immunology Group, International Centre for Genetic Engineering and Biotechnology, New Delhi, India; ^2^Drug Discovery Research Center, Translational Health Science and Technology Institute, Faridabad, India

**Keywords:** hypertrophy, E3 ligases, TRAFs, DUBs, ubiquitination, ubiquitin proteasome system

## Abstract

The ubiquitin proteasome system (UPS) plays an imperative role in many critical cellular processes, frequently by mediating the selective degradation of misfolded and damaged proteins and also by playing a non-degradative role especially important as in many signaling pathways. Over the last three decades, accumulated evidence indicated that UPS proteins are primal modulators of cell cycle progression, DNA replication, and repair, transcription, immune responses, and apoptosis. Comparatively, latest studies have demonstrated a substantial complexity by the UPS regulation in the heart. In addition, various UPS proteins especially ubiquitin ligases and proteasome have been identified to play a significant role in the cardiac development and dynamic physiology of cardiac pathologies such as ischemia/reperfusion injury, hypertrophy, and heart failure. However, our understanding of the contribution of UPS dysfunction in the plausible development of cardiac pathophysiology and the complete list of UPS proteins regulating these afflictions is still in infancy. The recent emergence of the roles of TNF receptor-associated factor (TRAFs) and deubiquitinating enzymes (DUBs) superfamily in hypertrophic cardiomyopathy has enhanced our knowledge. In this review, we have mainly compiled the TRAF superfamily of E3 ligases and few DUBs proteins with other well-documented E3 ligases such as MDM2, MuRF-1, Atrogin-I, and TRIM 32 that are specific to myocardial hypertrophy. In this review, we also aim to highlight their expression profile following physiological and pathological stimulation leading to the onset of hypertrophic phenotype in the heart that can serve as biomarkers and the opportunity for the development of novel therapies.

## Introduction

Cardiac hypertrophy is the second most common type of heart disease and has an incidence of 1 in 500 people worldwide (Jacoby et al., 2013). Cardiac hypertrophy is linked to thickening of primarily the left ventricle, leading to the reduction in pumping ability of the heart and cardiac dysfunction (Frey and Olson, 2003; Rohilla et al., 2012). Other characteristics associated with the disease are enlargement of cardiomyocyte with an augmentation in the protein synthesis and changes in the sarcomere organization (Carreño et al., 2006; Sequeira et al., 2014). Cardiac hypertrophy is primarily categorized as physiological or pathological and can be further classified as eccentric or concentric in response to volume and pressure overload, respectively (Shimizu and Minamino, 2016). Physiological hypertrophy is the compensatory response of the body to the normal changes such as during pregnancy or in athletes during training or any healthy exercise, with normal cardiac structure and organization, resulting in increased pumping capacity and myocardial mass. In contrast, pathological hypertrophy stems as a result of hypertension, some type of genetic mutation, valvular heart disease etc. and is characterized by the aggregation of collagen, enhancement of muscle mass, increase in myocyte death by apoptosis or necrosis and the reduction in systolic and diastolic function that ultimately leads to the heart failure (Maillet et al., 2013; Lyon et al., 2015). A multitude of factors such as thyroid disorders, weakening of heart muscles, abnormal heartbeat, protein deposition in heart, mutations in sarcomeric proteins, anemia etc., are known to contribute to the cardiac hypertrophy (Komuro, 2001; Niimura et al., 2002; Klein et al., 2005). Numerous signaling pathways are believed to be involved in leading toward hypertrophy for example, signals from hormones like insulin, thyroid and other mechanical forces leading to the activation of insulin-like growth factor-I (IGF-I)–phosphatidylinositol 3-kinase (PI3K)–Akt/protein kinase B (PKB) signaling, mammalian target of rapamycin (mTOR) pathway, calcineurin–nuclear factor of activated T cells (NFAT) pathway, mitogen-activated protein kinase (MAPK), tumor necrotic factor-alpha (TNF-α) and transforming growth factor-beta (TGF-β) (Lips et al., 2003; Wilkins and Molkentin, 2004; Dorn and Force, 2005; Heineke and Molkentin, 2006; Gupta et al., 2007).

Cardiomyocytes are the terminally differentiated cells possessing a very poor capacity of regeneration (Kikuchi and Poss, 2012). Therefore, it’s really important to maintain the protein quality control, which involves the recognition of damaged proteins for their degradation and replacement by newly synthesized proteins. Cardiac hypertrophy where protein synthesis has enhanced enormously, the regulatory check in terms of their timely degradation is equally important (Willis and Patterson, 2006). In this regard, a concept of Ubiquitin proteasome system (UPS) has emerged as a fascinating research area to treat the cardiac hypertrophy (Hedhli and Depre, 2009). In brief, the UPS is a post-translational mechanism that regulates the timely degradation of proteins (Lecker et al., 2006). The UPS is a three-cascade signaling pathway that involves ubiquitin-activating enzymes (E1), ubiquitin-conjugating enzymes (E2), and ubiquitin ligases (E3) (Scheffner et al., 1995). In the ubiquitination process, covalent attachment of C-terminal carboxyl group of ubiquitin to the amine of a lysine residue of protein occurs through an isopeptide bond. Different chains of ubiquitin with different linkages are formed such as K6, K11, K27, K29, K33, K48, and K63 depending on which of the seven available lysine residues in the ubiquitin molecule are being used (Akutsu et al., 2016). Ubiquitin chains are classified as linear, monoubiquitination, multimonoubiquitination, branched ubiquitination or mixed ubiquitin chain. Different chains are recognized by different proteins and targeted to different signaling pathway. K11 and K48 ubiquitin linkages target protein to the proteasomal degradation. Other types of linkages are involved in different function such as DNA repair, chromatin remodeling, endocytosis, kinase activation, intracellular trafficking and transcription regulation (Li and Ye, 2008). The major specificity of the UPS is chiefly governed by ∼700 E3 ubiquitin ligases (Metzger et al., 2012), which directs proteins for degradation by the 26S proteasome and by ∼100 deubiquitinating enzymes (DUBs; Nijman et al., 2005; Komander et al., 2009) that reverse the process of ubiquitination by the removal of ubiquitin chains. DUBs are known to associate with substrate adaptors, regulatory proteins and inhibitors modulating their function in cellular processes (Amerik and Hochstrasser, 2004; Reyes-Turcu et al., 2009). Extensive studies have established the pivotal role of the E3 ligases and DUBs in cell proliferation, differentiation and apoptosis (He et al., 2017; Gupta et al., 2018; Rape, 2018). With the UPS also comes the autophagy lysosome system for clearing the aberrant aggregate of proteins and damaged organelles (mitochondria) (Varshavsky, 2017). Accumulating literature evidence emphasizes a key role for cellular autophagy in maintaining homeostasis of cardiac myocytes (Parry and Willis, 2016). Induction of autophagy by rapamycin suppresses pressure overload induced cardiac hypertrophy in mice (Shioi et al., 2003; Mcmullen et al., 2004), whereas knockdown of autophagic genes such as Atg5 and Atg7 has shown to induce pressure overloaded cardiomyocyte hypertrophy (Nakai et al., 2007). However, the excess autophagic activity can exacerbate cardiac hypertrophy and may lead to heart failure, thus pointing at the controversial role of autophagy in cardiac hypertrophy (Li Z. et al., 2015). The role of autophagy in promoting or preventing cardiac hypertrophy varies with disease stage, severity and also with the different types of stress (Li et al., 2016).

The accumulation of ubiquitinated proteins in some of the cardiac diseases proposes the role of UPS dysregulation (Li and Wang, 2011). UPS is also known to significantly influence the maintenance of a balance of cardiac proteins both at steady state and in case of any type of stress (Beardslee et al., 1998). This balance is achieved by the continuous degradation and synthesis of the structural proteins such as sarcomere and also proteins involved in the regulation of different signaling pathways (Eble et al., 1999). The abundance of few of the E3 ligases and DUBs such as Atrogin-1, muscle ring finger protein-1 (MuRF1), A20, cylindromatosis (CYLD) have paved the way for the better understanding of the ubiquitin proteasome pathway in the heart (Arya et al., 2004; Li et al., 2007; Huang et al., 2010; Wang et al., 2015; Yu et al., 2017). Recently, for instance, an adenoviral overexpression of Atrogin-1 and MuRF1 E3 ligases suppresses the cardiac hypertrophy induced by phenylephrine treatment (Wertz et al., 2004; Maejima et al., 2014). These studies will provide novel insight into the E3s and DUBs operated signaling cascades and their distinct role in cardiac myopathies (Parry and Willis, 2016; Brown et al., 2017). Different components of UPS have been egressed out as significant drug targets in the treatment of cancer, immunodeficiencies, and neurodegenerative diseases (Paul, 2008; Shen et al., 2013; Pal et al., 2014; Upadhyay et al., 2017). Various studies have highlighted the role of the proteasome machinery in the cardiac diseases (Willis and Patterson, 2006; Zolk et al., 2006; Gilda and Gomes, 2017). Proteasome inhibitors are being used to treat cardiac diseases advocating them as a potential drug target (Drews and Taegtmeyer, 2014). Proteasome inhibition is shown to restrict the disease progression in the case of cardiac remodeling and pathological hypertrophy. In contrast, an increase of proteasome activities has been shown to improve cardiomyopathies and damage caused by oxidative stress (Li et al., 2011; Gilda and Gomes, 2017). However, the role and regulation by the UPS players are yet to be explored that can be targeted in the cardiovascular diseases. Therefore, futuristic studies on the E3s and DUBs to promote or prevent different substrates involved in hypertrophy are required further identified them to act either as biomarkers or as a potential therapeutic target. Here in this review, we have highlighted the role of the different E3 ligases and DUBs in regulating cardiac hypertrophy via different signaling cascades, further adding to the list as potential drug targets to attenuate or prevent the onset of cardiac hypertrophy.

## E3 Ligases Involved in Cardiac Hypertrophy

Ubiquitin ligases (E3s) are the largest class of UPS enzymes that are known to tag ubiquitin, a 76-amino acid moiety to the majority of substrates in eukaryotic cells and to infer specificity to the process (Ardley and Robinson, 2005; Berndsen and Wolberger, 2014). Three classes of the E3s are classified on the basis of mechanism they follow for transferring ubiquitin molecules from the E2 enzyme onto the substrate molecule: Really interesting new gene (RING), Homologous to E6AP C-terminus (HECT), and RING-between-RING (RBR) (Metzger et al., 2012; Spratt et al., 2014; Gupta et al., 2018). RING is the diverse and most abundant class of E3 ligases that exist either as monomers or oligomers. There are few RING E3s that exist as large multi-subunit complexes, comprising a catalytic RING finger protein, a protein from cullin family and a substrate adaptor protein such as F-box (Metzger et al., 2014).

### MDM2

**FIGURE 1:**
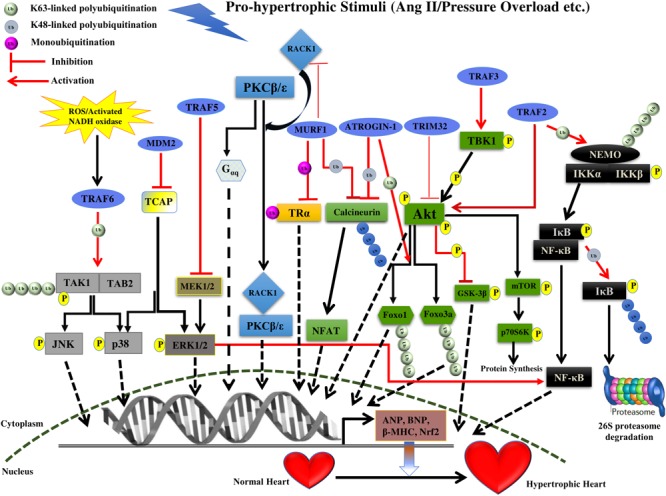
Summary of roles of different E3 ligases on the development of cardiac hypertrophy. The figure represents different pathways through which different E3 ligases of the UPS coordinates cardiac hypertrophic response. Different E3 ligases promoting/inhibiting the specific step in the signaling pathway are shown in the figure. E3 ligases are shown as blue ovals. The nucleus is shown at the bottom with nuclear membrane shown as a brown dashed line.

Multi-domain member of RING finger E3 ligase superfamily, MDM2 mediates ubiquitination of p53 led to inactivation of its transcriptional activities and resulting in its degradation thus regulating the vital cellular processes like cell cycle and apoptosis (Momand et al., 1992, 2000; Chen et al., 1994, 1996; Haupt et al., 1996, 1997; Honda et al., 1997; Kubbutat et al., 1997; Iwakuma and Lozano, 2003). The loss of MDM2 has a disastrous consequence, such that MDM2 deletion in mice resulting in their death as early as at blastocyst stage (Jones et al., 1995; Donehower and Lozano, 2009). However, the exact role of MDM2 in the heart specifically in the context of the development of hypertrophy remains understudied. MDM2 overexpression attenuates hypertrophy response in cardiac myocytes treated by the α-agonists phenylephrine or endothelin-1 although the effect of MDM2 does not depend on its ubiquitin ligase activity (Tian et al., 2006; Toth et al., 2006). Conditional cardiac-specific MDM2 gene knockout mice resulted in concentric cardiac hypertrophy followed by death within 7 days of tamoxifen treatment possibly as an outcome of the activation of p38 and ERK1/2 signaling pathways (Hauck et al., 2017; **Figure 1** and **Table 1**). MDM2 thus having a dual function of being antiapoptotic as well as of preventing hypertrophy in cardiomyocytes advocates the idea of MDM2 serving as a novel cardiac gene therapy target to downregulate both apoptosis and pathologic cardiac hypertrophy. However, the mechanistic details of MDM2 inhibiting particularly, the cardiac hypertrophy, remains unsettled.

**Table 1:** List mentioning the functions of E3 ligases and DUBs involved in cardiac hypertrophy.

Name	Signaling pathway
MDM2	Attenuation of pathological cardiac hypertrophy via inhibition of the p38 and ERK1/2 pathway
MuRF1	Inhibition of pathological hypertrophy via inhibiting protein kinase C (PKC) mediated signaling and calcineurin-NFAT pathway. Inhibition of T3-induced physiological hypertrophy by the monoubiquitination of the TRα receptor in the heart
MuRF2	Induction of cardiac hypertrophy in MuRF1/MuRF2 double knockout mice
MuRF3	Induction of cardiac hypertrophy in MuRF3/MuRF1 double knockout mice by the accumulation of β/slow MHC protein
Atrogin-1	Suppression of cardiac hypertrophy via inhibition of calcineurin-NFAT pathway by polyubiquitination and degradation of the calcineurin A together with SCF complex and through K63-linked polyubiquitination of Foxo1 and Foxo3a
TRIM32	Attenuation of pressure overload-induced hypertrophy via inhibition of the Akt-GSK3β-mTOR-p70S6K pathway
TRAF2	Induction of pathological cardiac hypertrophy via activation of the NF-κB and Akt/GSK3β pathway
TRAF3	Induction of pathological cardiac hypertrophy via activation of the Akt-GSK3β-mTOR-p70S6K pathway
TRAF5	Attenuation of cardiac hypertrophy via inhibition of the MEK-ERK1/2 pathway
TRAF6	Induction of cardiac hypertrophy via activation of the TAK1-JNK1/2-p38 pathway
USP4	Inhibition of cardiac hypertrophy via inhibition of the TAK1-JNK1/2-p38 pathway
USP18	Inhibition of cardiac hypertrophy via inhibition of the TAK1-JNK1/2-p38 pathway
USP14	Induction of cardiac hypertrophy via activation of the GSK-3β phosphorylation
CYLD	Induction of cardiac hypertrophy via inhibition of the ERK, p38/AP-1, and c-Myc pathway
A20	Suppression of cardiac hypertrophy via inhibition of the TAK1-JNK1/2-p38 pathway

### MuRFs

MuRF is the muscle-specific RING finger family of E3 ligases, expressed particularly in cardiac and skeletal muscles. These proteins are characterized as RBCC or TRIM class of proteins due to the characteristic RING, B-Box and a coiled-coil domain (Ikeda and Inoue, 2012). MuRF family specific motif (MFC-motif) is the additional domain between RING and the B-Box that is present in the MuRF class of proteins (Mrosek et al., 2008). MuRF class of proteins MuRF 1, 2, and 3 are known to be specifically expressed in striated muscles. They can create complexes as homo or heterodimers and regulate cardiac development and contractility.

MuRF1, one of the members of the MuRF E3 ligases has been identified to maintain the balance of hypertrophic and anti-hypertrophic signaling within myocytes. This balance is achieved by inhibition of the protein kinase C (PKC) mediated signaling in myocytes. PKCs are an important trigger of cardiac hypertrophy and activation of PKC𝜀 leads to hypertrophy in two ways either by G_αq_ overexpression or by its association with the receptor for activated C kinase 1 (RACK1), which ultimately leads to cardiac hypertrophy (**Figure 1**). RACK1 is a crucial scaffold protein that acts as a shuttling protein across the cell, stabilizing protein activity and in many cases of PKC, it is known to stabilize it at the membrane to phosphorylate subsequent players (Slager et al., 2008; Adams et al., 2011). PKCβII is known to develop hypertrophy by increasing troponin I phosphorylation. MuRF1 interaction with RACK1 and inhibition of PKC𝜀 translocation accelerated by phenylephrine leading to inhibition of hypertrophy (**Table 1**). This suggests modulation of switch between PKCβII-RACK1 and PKC𝜀-RACK1 interactions. However, this study has not highlighted the E3 ligase activity of MuRF1, as the levels of PKC and RACK1 were not affected but there might be other upstream and downstream effectors to be modulated by ligase activity of MuRF1 (Arya et al., 2004).

Interestingly MuRF1 also regulates the Tri-iodothyronine (T3), an active form of thyroid hormone (TH) that maintains homeostasis of heart. MuRF1 is shown to inhibit the T3-induced physiological cardiac hypertrophy. MuRF1 monoubiquitinates TRα receptor in the heart, resulting in enhancement of its affinity to centrosome-associated protein 350 (CAP350), which seizes TRα-Ub in nuclear compartments and ultimately leads to a reduction in its activity. This study provides a novel role of TRα in regulating cardiac hypertrophy by non-canonical ubiquitination (Wadosky et al., 2016; **Table 1**). It has also been noted that downregulation of MuRF1 shown to enhance cardiac hypertrophy in case of pressure overload. This is achieved by MuRF1 mediated polyubiquitination and degradation of calcineurin A leading to suppression of the calcineurin-NFAT pathway (Maejima et al., 2014; **Figure 1** and **Table 1**). It will be interesting to explore the molecular mechanism responsible for downregulation of MuRF1 in case of cardiac hypertrophy.

Some of the functions of MuRF1 and MuRF2 role is redundant in cardiac development. Both of them are known to interact with titin, troponin I and troponin T proteins (Witt et al., 2005). It is found that MuRF1/MuRF2 double knockout mice develop cardiac hypertrophy while lack of three out of four, MuRF1 and MuRF2 alleles were identical to wild-type mice phenotypically (Rodriguez et al., 2009). An extensive study on MuRFs has also identified higher rare variants of MuRF1 and MuRF2 in hypertrophic cardiomyopathy patients as compared to control group (Su et al., 2014). Certainly, these mutations are associated with impaired protein degradation in cardiac myocytes, leading to accumulation of cardiac hypertrophy regulators. These studies suggest the role of MuRF2 in cardiac hypertrophy and further studies will pave the way for better understanding.

MuRF3 is identified to interact with the microtubules and shown to target γ-filamin and four and a half LIM protein 2 (FHL2) to ubiquitin proteasome mediated degradation (Fielitz et al., 2007b). MuRF3 and MuRF1 double knockout mice develop hypertrophic cardiomyopathy. The accumulation of β/slow MHC protein and its breakdown products in hearts of double knockout compared with wild-type could be observed, revealing the role of the UPS dependent turnover of sarcomeric proteins (Fielitz et al., 2007a). There is a need to study in detail the mechanism of degradation of sarcomeric proteins as this could be beneficial in the design of MuRF inhibitors.

### Atrogin-1

Atrogin-1 is an F-box protein that interacts with Skp1, Cul1, and Roc1 to form the SCF complex of multicomponent ubiquitin ligase family. F-box proteins are known to act as an adaptor, binding to specific substrates molecules and mediate ubiquitin-dependent degradation (Willems et al., 2004). Atrogin-1 lacks the leucine-rich regions and WD-40 repeats domain, which are present in other F-box proteins to mediate the protein-protein interaction (Gomes et al., 2001). Atrogin-1 interacts with calcineurin A in cardiac myofibril and together with SCF complex it polyubiquitinates and degrades the calcineurin A leading to the inhibition of cardiac hypertrophy (Wertz et al., 2004). Atrogin-1 inhibits the calcineurin-NFAT signaling pathway, however, molecular mechanistic details and other downstream mediators still need to be unraveled. Interestingly Atrogin-1 also inhibits cardiac hypertrophy via the Akt-dependent pathway, where it has no effect on Akt, but it coactivates Forkhead family of transcription factors, which is downstream of Akt (**Figure 1** and **Table 1**). Atrogin-1 mediates K63-linked polyubiquitination of Foxo1 and Foxo3a, which serve as a signal for recruitment of co-activators or co-repressors, and possibly other such downstream players that still need to be identified (Li et al., 2007). Further, in-depth characterization of Atrogin-1 can provide an understanding of how these F-box proteins are activated and involved in diverse pathways that ultimately led to the suppression of cardiac hypertrophy.

### TRIM32

TRIM32 is the E3 ligase with the common tripartite motif (RING, B-Box and coiled-coil domain) of TRIpartite Motif (TRIM) family protein but also have an additional six NHL repeats at C terminal (Tocchini and Ciosk, 2015). The level of TRIM32 was decreased in cardiomyocytes in case of hypertrophic stress. The expression level of hypertrophic markers atrial natriuretic peptide (ANP), brain natriuretic peptide (BNP) and beta-myosin heavy chain (β-MHC) was increased in TRIM32-deficient cardiomyocytes. TRIM32 overexpression is shown to attenuate pressure overload-induced hypertrophy and this is suggested by the decrease in the ratios of heart weight to body weight (HW/BW), the ratios of HW to tibia length (HW/TL) (Chen et al., 2016). TRIM32 deficient was shown to increase the phosphorylation of Akt and subsequent players [e.g., glycogen synthase kinase 3β (GSK3β), mTOR, and p70S6K], and activate Akt-GSK3β-mTOR-p70S6K signaling pathway (**Figure 1** and **Table 1**). This study suggested that TRIM32 provide cardioprotection against cardiac hypertrophy. Futuristic studies on additional players in Akt pathway will certainly provide more insight into the signaling pathway regulated by TRIM32.

### Tumor Necrosis Factor Receptor-Associated Factors (TRAFs)

Tumor necrosis factor receptor-associated factors (TRAFs) are adapter proteins recognized as a signal mediator for the tumor necrosis factor receptor (TNFR), the Epstein–Barr virus protein LMP1, and interleukin-1 receptor/Toll-like receptor superfamily members. TRAF subfamily consists of seven isoforms (TRAF1-7) that are multidomain proteins, containing a zinc finger motif, a coiled-coil (leucine zipper) motif, C-terminal TRAF domain (except TRAF7), and an N-terminal RING domain (except TRAF1) containing ubiquitin ligase activity. TRAFs are critically involved in vital processes in the cell including the innate and adaptive immune response, differentiation, apoptosis, and proliferation and involve in activating various signaling pathways such as NF-κB, and JNK (Nakano et al., 1996, 1997; Inoue et al., 2000; Bradley and Pober, 2001). However, our knowledge regarding the role of TRAF proteins in the heart is still limited. Recently, the involvement and therapeutic potential of TRAF proteins in the onset of cardiac myopathies have been explored to gain more insights.

#### TRAF2

TRAF2 role has been well documented in the regulation of various biological activities such as immune and inflammatory responses and apoptosis (Jono et al., 2004; Ermolaeva et al., 2008; Shih et al., 2011; Huang et al., 2014). Though TRAF2 is a scaffold protein that is involved in a number of signal transduction pathways including MAPK, NF-κB, and JNK, that prominently contribute to the hypertrophic phenotype of the heart (Gupta and Sen, 2005; Gupta et al., 2005; Huang et al., 2014). However, the precise contribution of TRAF2 in the development of adult mammalian cardiac hypertrophy is somewhat unclear.

Transgenic mice overexpressing TRAF2 driven by MHC promoter (MHC-TRAF2_HC_ demonstrated the symptoms of progressive cardiac hypertrophy with increased myocardial fibrosis even after 12 weeks (Divakaran et al., 2013). In brief, the study illustrated an increase in LV end diastolic dimension n with corresponding increase in radius/wall (r/h) thickness ratio by the end of 12 weeks in MHC-TRAF2_HC_ transgenic mice as compared to controls. Mouse expressing TRAF2 also showed a 4.4-fold increase in collagen content in the extracellular matrix, thus depicting the induction of myocardial fibrosis. A significant increase in nuclear factor-κB (NF-κB) activation, as well as the level of downstream molecules (p50, RelB, and p52 subunits), was observed from 4 to 12 weeks (Divakaran et al., 2013; **Figure 1** and **Table 1**). Increasing amount of literature suggests the vital role played by NF-κB signaling pathway in regulating the process of cardiac hypertrophy and heart failure (Purcell et al., 2001; Li et al., 2004; Freund et al., 2005; Zelarayan et al., 2009; Santos et al., 2010; Gordon et al., 2011; Leychenko et al., 2011). However, the exact contribution of NF-κB activation in the induction of cardiac hypertrophy when TRAF2 is overexpressed needs to be further verified.

Interestingly, Huang et al. (2014) illustrated the activation of Akt/GSK3 signaling pathway on TRAF2 overexpression in the hypertrophic heart. In this study, a significant up-regulation of the endogenous TRAF2 and fetal genes (such as ANP and BNP) expression in mice failing hearts was observed 8 weeks after the TAC treatment. Over-expression of TRAF2 by Ad-TRAF2 infection in cultured isolated cardiomyocytes leads to their enhanced hypertrophy in response to Ang II treatment *in vitro*, whereas, decreased TRAF2 expression by Ad-shTRAF2 infection attenuated the hypertrophy. Similar to the study by Divakaran et al. (2013), transgenic mice expressing TRAF2 cDNA by α- MHC promoter, demonstrated significant cardiac hypertrophy after 4 weeks in comparison to wild-type in response to TAC stimulation. Transgenic mice demonstrated an increase in HW/BW, HW/TL ratios, LV chamber dimension, wall thickness and the individual cardiomyocytes cross-sectional area in addition to the overexpression of fetal genes (ANP, BNP, and β-MHC; Huang et al., 2014). Phosphorylation levels of Akt and its downstream targets including GSK3β were observed to be much increased in response to pressure overload both *in vivo* and *in vitro* (Huang et al., 2014; **Figure 1** and **Table 1**). Akt signaling pathway is known for its role in cardiac hypertrophy (Condorelli et al., 2002; Predmore et al., 2010; Chen et al., 2013; Maillet et al., 2013; Jiang et al., 2015), however, the exact mechanism by which TRAF2 specifically activating Akt pathway is not clear.

It has been shown that the development of cardiac hypertrophy is an outcome of the activation of many signaling events including MAPK, NF-κB, calcineurin/NFAT cell signaling pathways (Purcell et al., 2001; Frey and Olson, 2003; Gordon et al., 2011). Previous studies suggest that Akt regulates NF-κB signaling by inducing phosphorylation of inhibitor of κB (IκB) and its subsequent degradation by proteasomes (Ozes et al., 1999; Gustin et al., 2004). Therefore, it may be possible that as a critical protein component of NF-κB signaling, TRAF2 modulates NF-κB signaling complex through first activating the Akt signaling that ultimately resulted in cardiac hypertrophy. However, futuristic experimental reports will establish an exact mechanism followed by TRAF2 in regulating the development of cardiac hypertrophy.

TRAF2 may also play a role in the activation of autophagy in the early phase of ER stress. An active IRE1 on ER membrane stimulates JNK pathway using its kinase activity via recruitment of TRAF2 that resulted into the activation of autophagosomes (Urano et al., 2000; Ogata et al., 2006). Recent studies have thrown open the potential regulation of cardiomyocyte hypertrophy by cardiac autophagy mechanism. However, the exact mechanism by which cardiac autophagy and hypertrophic response are linked needs to be further explored (Li et al., 2016).

#### TRAF3

Similar to other TRAFs, TRAF3 regulates the activities of several signaling pathways, for example, TRAF3 degradation in B cells induces activation of MAPK and NF-κB signaling pathways (Matsuzawa et al., 2008; Vallabhapurapu et al., 2008), while, binding of TRAF3 to PI3K promotes activation of CD40-associated Akt pathway (Fang et al., 2014). Role of MAPK, NF-κB, and Akt pathways is well documented in the development of hypertrophic response in heart (Purcell et al., 2001; Condorelli et al., 2002; Frey and Olson, 2003; Li et al., 2004; Predmore et al., 2010; Gordon et al., 2011), however, the exact role of TRAF3 in the development of the disease in response to hypertrophic stimuli is not well documented.

Jiang et al. (2015) identified TRAF3 as a key regulator of hypertrophic response against pressure overload. TRAF3 protein levels were increased significantly in hypertrophied mice and failing human hearts in comparison to normal ones. In response to pressure overload by aortic banding (AB), TRAF3-knockout mice demonstrated significantly decreased cardiac hypertrophy after 4 weeks as depicted by individual cardiomyocyte cross-sectional area along with reduced cardiac fibrosis and preserved cardiac function as seen in reduction in HW/BW, HW/TL and lung weight/BW (LW/BW) ratios. Whereas, transgenic mice overexpressing TRAF3 showed an increase in cardiac hypertrophy after 4 weeks as indicated by significant increase in cardiomyocyte size and fibrosis as well as higher HW/BW, HW/TL, and LW/BW ratios and increased mRNA level of cardiac fetal genes (ANP, BNP, and β-MHC). Consistent with these results, overexpression of TRAF3 in isolated neonatal rat cardiomyocytes (NRCMs) showed a significant hypertrophic response *in vitro* with enhanced β-MHC and ANP mRNA levels when treated with angiotensin II– or phenylephrine, whereas, TRAF3 knockdown inhibited cardiomyocyte hypertrophy on similar treatment. Study demonstrated the increase in phosphorylation of Akt and downstream molecules (e.g., GSK3β, mTOR and p70S6K), both in TRAF3-TG mice as well as NRCMs overexpressing TRAF3 in response to pressure overload (Ang-II or phenylephrine), that was much low in TRAF3-KO mice and NRCMs expressing the TRAF3 deletion mutant (Jiang et al., 2015). In addition, Akt-specific inhibitor MK-2206 showed inhibition in the hypertrophy caused by TRAF3 overexpression *in vitro*. Inhibition of mTORC1 by rapamycin treatment also reversed cardiac hypertrophy induced by Akt overexpression (Shioi et al., 2000; Shiojima et al., 2005). Taken together, TRAF3 positively regulates the Akt-GSK3β-mTOR-p70S6K signaling pathway in the development of pathological cardiac hypertrophy (Jiang et al., 2015; **Figure 1** and **Table 1**).

Co-immunoprecipitation and mutation experiments showed the binding of TRAF3 to TANK-binding kinase 1 (TBK1) that results in enhanced TBK1 phosphorylation which further activates Akt via direct phosphorylation at both S473 and T308 (Joung et al., 2011; Xie et al., 2011; **Figure 1**). In addition, knockdown of TBK1 almost prevented the cardiac hypertrophy by TRAF3 overexpression in response to Ang II. Therefore, the TRAF3-TBK1-Akt signaling pathway is believed to be involved in the development of cardiac hypertrophy (**Table 1**). Thus, targeting TRAF3–TBK1 binding and inhibiting resultant Akt signaling might be an effective strategy to treat cardiac hypertrophy.

#### TRAF5

TRAF5 has been identified as a positive regulator of various TNFR superfamily receptors and shown to involved in the innate immune response following viral infection. It has been shown to be involved in JNK and p38 MAPK activation in cultured macrophages, fibroblasts and in the aortas of TRAF5-deficient animals (Nakano et al., 1999; Shirakata et al., 2001; Kraus et al., 2009; Missiou et al., 2010). Moreover, the role of TRAF5 in TNF-induced NF-κB activation in murine embryonic fibroblasts has also been documented (Tada et al., 2001). Bian et al. (2014) demonstrated that TRAF5 is involved in the development of pressure-overload-induced cardiac hypertrophy. Study involving TRAF5 gene knockout (KO) mice portrayed substantially aggravated cardiac hypertrophy, cardiac dysfunction and fibrosis confirmed by an increase in HW/BW, LW/BW, and HW/TL ratios and also in ANP, BNP, and β-MHC expression level, as well as myocyte cell enhancements, compared to wild-type (WT) mice 4 weeks following TAC surgery (**Table 1**). In contrast, *in vitro* overexpression of TRAF5 in isolated cardiomyocytes exhibited a marked reduction in the expression of hypertrophic markers on Ang II stimulation. Furthermore, TRAF5 deficiency showed an increase in the phosphorylation level of NF-κB, MEK and ERK1/2 in response to TAC in KO mice in comparison to controls, whereas, TRAF5 overexpression in cultured H9c2 cardiomyocytes showed decrease in NF-κB, MEK, and ERK1/2 phosphorylation level after Ang II treatment (Bian et al., 2014; **Figure 1** and **Table 1**). However, TRAF5 role pertaining to the activation of NF-κB seems to be controversial. Several studies advocate the involvement of TRAF5 in NF-κB activation (Nakano et al., 1996; Akiba et al., 1998; Mizushima et al., 1998), however, study by Bian et al. (2014) showed the activated NF-κB in the hearts of TRAF5-deficient animals under pressure overload. Moreover, Tada et al showed TNF-induced NF-κB translocation not to be affected in TRAF5 KO mice but only in double knock-out (DKO) mutant of TRAF2 and TRAF5 in mouse embryo fibroblasts (Tada et al., 2001).

The previous study by the same group proved that ERK1/2 is responsible for the activation of NF-κB (Shen et al., 2010; **Figure 1**). Thus, the pressure overload-induced pathologic cardiac hypertrophy on account of TRAF5 deficiency is most likely a result of the activation of ERK1/2-dependent NF-κB and MEK-ERK1/2 signaling. However, future studies will establish the precise mechanism of TRAF5 regulating the MEK-ERK1/2 pathway.

#### TRAF6

TRAF6 protein found in most tissues including skeletal and smooth muscles, endothelial cells, adipose tissues, B-cells, macrophages and is also prominently expressed in the cardiomyocytes and cardiac fibroblast indicative of its potential role in cardiac inflammation and hypertrophy as well (Cao et al., 1996; Gu et al., 2012; Chen et al., 2015; Dong et al., 2015; Iwata et al., 2015; Ji et al., 2016; Abdullah et al., 2017). TRAF6 interacts with a range of targets including various membrane receptors, adaptor proteins, and intracellular kinases through TRAF6 binding motifs present in the substrates making it a multifaceted mediator of signaling pathways (Sebban-Benin et al., 2007; Mu et al., 2011). TRAF6 is believed to take part in TLR and NF-κB signaling and have critical roles in tissue homeostasis and inflammatory processes (Lomaga et al., 1999; Wu and Arron, 2003; Newton and Dixit, 2012; Luong et al., 2013).

Ji et al. (2016) reported the levels of TRAF6 protein significantly upregulated in hypertrophic and failing human hearts, along with the re-expression of fetal genes (ANP and β-MHC). TRAF6 protein expression levels were found progressively increasing from weeks 2–8 in experimental mice in response to pressure overload (e.g., AB surgery) as compared to sham-operated controls. Similarly, isolated neonatal rat cardiomyocytes (NRCMs) exhibited elevated TRAF6 level at 48 h in response to angiotensin II or phenylephrine treatment compared with controls treated with phosphate-buffered saline (PBS) (Ji et al., 2016). Several studies have identified intracellular oxidative stress to activate TRAF6 (Matsuzawa et al., 2005; Fujino et al., 2007; Imai et al., 2008). Hydrogen peroxide (H_2_O_2_) stimulus and treatment with ROS scavengers including apocynin (APO) and *N*-acetyl-cysteine (NAC) identified the upregulation of ROS and NADPH oxidase expression to be largely responsible for the progressive induction of TRAF6 expression during the development of pathological cardiac hypertrophy both in experimental mice (TRAF6-Tg or TRAF6-CKO) and in isolated NRCMs (Ji et al., 2016; **Figure 1**).

Experiments using transgenic mice overexpressing cardiomyocyte-specific TRAF6 illustrated a notable increase in hypertrophy and fibrosis after AB surgery or in response to neurohormonal stimuli (for example, Ang II) as shown by significant increase in HW/BW, LW/BW and HW/TL ratios and elevated mRNA levels of pathologic hypertrophy markers (such as ANP, BNP, and β-MHC) in comparison to controls (Ji et al., 2016) (**Table 1**). On similar lines, the *in vitro* studies involving NCRMs infected with adenovirus harboring TRAF6 cDNA (AdTraf6) showed notably increased cardiac hypertrophy and increased ANP and β-MHC levels after 48 h on similar induction with Ang II or PE as compared to controls. Whereas, the downregulation or complete deletion of TRAF6 alleviated cardiac hypertrophy and cardiac dysfunction along with a decrease in expression levels of hypertrophy markers (Ji et al., 2016).

Regulation of pathologic cardiac hypertrophy by TRAF6 is possible via regulating TAK1-JNK1/2-p38 signaling pathway. TRAF6 overexpression both *in vivo* and *in vitro* showed the significant elevation in JNK1/2, p38 and upstream TAK1 phosphorylation level and their activation in response to Ang-II or pressure overload via TRAF6 auto-ubiquitination (Ji et al., 2016; **Figure 1**). Mechanistically, TRAF6 activation induced by ROS production and activation of NADPH oxidase triggers the binding of TRAF6 to TAK1 mediated by recruitment of adapter protein TAB2. Binding of TRAF6 to TAK1 results in K63-linked polyubiquitination and increased phosphorylation of TAK1 that further leads to the activation of the downstream JNK1/2 and p38 signaling (Takaesu et al., 2000; Kanayama et al., 2004; Xia et al., 2009; Ji et al., 2016; **Figure 1** and **Table 1**). Overexpression of activated TAK1 is sufficiently adequate to induce cardiac dysfunction and hypertrophy in response to pressure overload (Zhang et al., 2000; Watkins et al., 2012). Deleting or mutating (C70A mutant) TRAF6 ring domain (with ubiquitin ligase activity) inhibits both K63 ubiquitination of TAK1 and activation of p38 and JNK signaling, demonstrating the importance of TRAF6 E3 ligase activity in inducing pathologic cardiac hypertrophy (Cao et al., 2015; Ji et al., 2016). Another study demonstrated that mice with cardiac-specific deletion of TAK1 develops spontaneous cardiac remodeling and heart failure (Li et al., 2014), however, inducible transgenic mouse model expressing active TAK1ΔN mutant revealed the contrasting effect on heart failure progression after myocardial infarction. Thus, further studies are needed to determine the biological effect of timing and extent of TAK1 activation in the heart. Further, how post-translational activation of TRAF6 leads to activation of E3 ligase activity needs to be further explored. Overall, TRAF6 plays a critical role in the induction of cardiac hypertrophy and may prove to be a potential therapeutic target.

## DUBs Involved in Hypertrophy

Deubiquitinating enzymes (DUBs) are proteases that are imperative for the removal of ubiquitin chains resulting in the reversal of signaling or rescue of protein from degradation, or the recycling of ubiquitin for ubiquitin homeostasis (Wilkinson, 2009). The mechanism of action of DUBs involves nucleophilic attack on the carbonyl group of Ub-substrate isopeptide bond. They are generally categorized into five classes -aspartic, metallo, serine, threonine, and cysteine proteases, depending on the residues involved in the catalytic activity. Cysteine proteases are further classified into four subclasses – ubiquitin-specific protease (USP), ubiquitin C-terminal hydrolase (UCH), otubain protease (OTU), and machado-joseph disease protease (MJD). Metallo-proteases are JAMM (JAB1/MPN/Mov34 metalloenzyme) proteases, which employ metal ions for catalysis (Komander et al., 2009; Reyes-Turcu et al., 2009).

### Ubiquitin-Specific Protease (USP)

Ubiquitin-specific protease is the largest class of deubiquitinating enzyme, predicted to have 56 members of DUBs. The domain length of USP varies from 300 to 800 amino acids; most of them are parted by unrelated sequences. They also contain other domains such as ubiquitin-associated (UBA) domains, ubiquitin-interacting motifs (UIF) and zinc finger (ZnF UBP). The USP fold comprises of three sub-domains: the finger, palm, and thumb that resembles a right hand. The catalytic domain of USP contains two cysteines and a histidine box responsible for the catalytic activity and lies at the interface between palm and thumb. The mechanism of action involves the deprotonation of catalytic cysteine carried out by a histidine residue, which is polarized by an aspartic acid residue (Komander, 2010).

#### USP4

**FIGURE 2:**
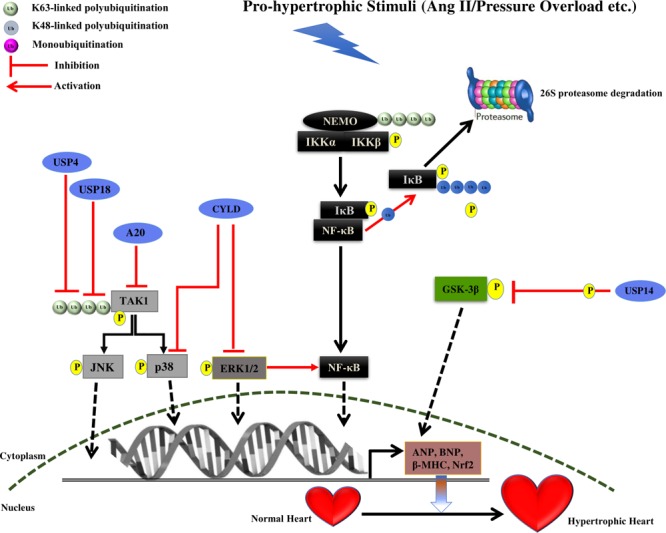
Summary of roles of different DUBs on the development of cardiac hypertrophy. The figure represents different pathways through which different Deubiquitinating enzymes (DUBs) of the UPS coordinates cardiac hypertrophic response. Different DUBs promoting/inhibiting the specific step in the signaling pathway are shown in the figure. DUBs are shown as blue ovals. The nucleus is shown at the bottom with nuclear membrane shown as a brown dashed line.

USP4 is known to expressed in heart and its levels were observed to be reduced in murine hypertrophied models and human failing hearts. In USP4 knockout mice models, an exacerbated hypertrophic growth and cardiac dysfunction were observed. It was also inferred by echocardiographic measurements that USP4 knockout promoted ventricular dilation and contractile dysfunction. Furthermore, the enhancement of collagen I, collagen III and connective tissue growth factor and reduced levels of sarcoplasmic reticulum Ca^2+^ ATPase (Serca2a) were also observed in USP4 knockout mice following pressure overload, suggesting interstitial and perivascular fibrosis. In parallel, the regaining of USP4 level protected the heart from hypertrophy. USP4 was identified to deubiquitinates transforming growth factor beta (TGF-beta)-activated kinase 1 (TAK1), resulting in suppressing of its autophosphorylation and consequent phosphorylation of c-Jun N-terminal kinase1/2 (JNK1/2) and p38 (**Figure 2** and **Table 1**). This leads to hindrance in the TAK1-JNK1/2-p38 signaling accountable for hypertrophy (He et al., 2016). USP4 (C311A) mutation did not inhibit the TAK1 polyubiquitination, and phosphorylation of TAK1, =JNK1/2, and p38 in angiotensin treated myocytes. This study suggests the crucial role of deubiquitinating activity of USP4 in hypertrophy. Further validation of TAK1 as the key mediator of hypertrophy was done by injecting an inhibitor of TAK1, 5z-7-oxozeaenol following AB surgery. It leads to the suppression of phosphorylation of TAK1 and subsequently reversed the hypertrophy in USP4 knockout mice. It was further noticed that restoration of the USP4 level is a potential strategy for overruling cardiac hypertrophy. Further, the study of the USP4 role in apoptosis-mediated hypertrophy would provide more elucidation in the disease.

#### USP18

USP18 expression in heart and its role in NF-κB and TAK1 signaling suggest its role in cardiac pathophysiology. Overexpression of USP18 was shown to inhibit cardiac hypertrophy while its depletion amplified the hypertrophic response. *In vitro* studies showed that USP18 knockout reduces the mRNA level of fetal genes such as ANP, BNP, and β-MHC, suggesting the protection of cardiac hypertrophy. Furthermore, USP18 knockout enhances the HW/BW ratio, the HW/TL ratio, and the HW/LW ratio after AB surgery. Experimental echocardiographic measurements recorded enlarged left ventricular end-diastolic and end-systolic dimensions and reduced ejection fraction in case of USP18 knockout mice in comparison to wild-type after subjection to AB surgery. The pathological fibrosis was also observed in AB surgery mice with the significant increase in fibrotic markers. Moreover, cardiac-specific overexpression of USP18 protected the heart against hypertrophy and fibrosis induced by pressure overload. Further, USP18 knockout mice showed the level of phosphorylated TAK1 was higher as compared to wild mice after AB surgery, implying the effect of USP18 in inhibition of the TAK1-JNK1/2-p38 activation (**Figure 2** and **Table 1**). USP18 was shown to deubiquitinates TAK1, leading to its decreased phosphorylation in case of cardiac hypertrophy (Ying et al., 2016; **Figure 1**). The TAK1 activity was also inhibited with its inhibitor 5z-7-oxozeaenol, resulting in reversion of hypertrophy due to USP18 knockout in subjection to pressure overload. Contradictorily, USP18 levels were exalted in hypertrophic murine models and human dilated hearts, due to two main reasons. First, increased hypertrophic levels in response to pathogenic stimuli and second, USP18 levels are seen to increase during the macrophage proliferation and monocyte activation. USP18 is shown to play a role in interferon signaling suggesting its role in inflammation. Thus, further investigation of the role of the USP18 in inflammation in cardiac hypertrophy protection is another aspect that can be looked upon.

#### USP14

USP14 is a DUB of the 19S proteasome subunit. USP14 levels were seen to be high in hypertrophied heart. When the deubiquitinating activity of USP14 was inhibited, that resulting in a decrease in GSK-3β phosphorylation (Liu et al., 2016; **Figure 2** and **Table 1**). Further studies in the identification of downstream partners would provide more insight into the mechanism.

#### Cylindromatosis (CYLD)

Cylindromatosis is known to have the conserved USP domain with catalytic triad and the thumb-palm cleft. The third fingers subdomain, an extension of palm subdomain is reduced in size in CYLD as compare to other USP. The three short strands (β4, β5, and β6) at the tip of the USP fingers is also absent in CYLD (Komander et al., 2008). CYLD is known to inhibit the NF-κB signaling by removing K63-linked polyubiquitin chain from NF-kappa-B essential modulator (NEMO) (Kovalenko et al., 2003; Trompouki et al., 2003). Considering its similarity to A20, it is considered to work as a suppressor of cardiac pathology. However, CYLD is known to act as a mediator of cardiac dysfunction and its levels are upregulated during the earlier stage of transverse aortic arch constriction (TAC) induced adaptive cardiac hypertrophy. CYLD is known to intercede cardiac maladaptive remodeling and dysfunction by increasing myocardial oxidative stress by deactivating the MAPK/Activator protein 1 (AP-1) and c-Myc pathways that are responsible for nuclear factor (erythroid-derived 2)-like 2 (Nrf2) expression, which is an important transcription factor of the antioxidant defense system. CYLD also enhances oxidative stress by inhibiting the activation of the extracellular signal-regulated kinase (ERK), p38/AP-1 and c-Myc pathways, ensuring Nrf2 downregulation (Wang et al., 2015). A new CYLD-Nrf2 axis in modulating cardiac dysfunction is highlighted, however, further study on CYLD deubiquitination mechanism in identifying one of the substrates in this pathway will bring more insight into this signaling. Future study on downstream effector molecules of Nrf2 and other factors or signaling pathways responsible for oxidative stress will provide more illumination on the role of oxidative stress contributing to cardiac hypertrophy.

The role of CYLD is also shown in the autophagic pathway, which further regulates the cardiovascular disease. Selective autophagy is mediated by cytosolic sensor receptors p62, HDAC, and NBR1 and is responsible for the destruction of large amount of aggregates of ubiquitinated proteins (Willis et al., 2010). CYLD is known to regulate selective autophagy by controlling the interaction with p62 directly and by inactivating HDAC6 (Yan et al., 2013). CYLD is also known to interact with other autophagic regulators such as Beclin-1, HMGB1, and HO1 (Wooten et al., 2008; Tang D. et al., 2010). Future work on autophagy and connecting link with cardiovascular disease will provide a better understanding.

### Otubain-Specific Proteases (OTU)

This class of DUB family includes about 15 members where domain length varies from 150 to 200 amino acids. They also contain additional domains such as UIM, UBA, and ubiquitin-like folds (UBL). The active site consisting of a catalytic triad where cysteine and histidine are structurally conserved and the third amino acid is different (Wolberger, 2014).

#### A20

A20 is the OTU family of cysteine protease DUB and contains an N-terminal OTU domain and seven C-terminal zinc-fingers (ZF) motifs (Verhelst et al., 2014). A20 performs a dual role of both deubiquitination and ubiquitination and is known to inhibit the NF-κB signaling by removing K63-linked ubiquitination and by binding to linear ubiquitination (Bellail et al., 2012; Verhelst et al., 2012). A20 function in cardiac hypertrophy was investigated by its forced expression in the heart. A20 was shown to decrease cardiac hypertrophy by reducing the expression of ANP, BNP, and β-MHC proteins. To elucidate the mechanism of action, A20 overexpression was carried out in transgenic mice leading to suppression of the p38, JNK, and TAK1 activation. Thus, A20 protect against hypertrophy by inhibiting the TAK1-JNK1/2-p38 signaling (Huang et al., 2010). More study on the A20 mechanism of action, whether it is playing a role as an E3 ligase or DUB in this signaling could provide more clarification and give a novel perspective of the A20 role in cardiac hypertrophy.

Hypertrophy also result in cardiac fibrosis by the accumulation of collagen, A20 was shown to inhibit the collagen synthesis and fibrosis by inhibiting Smad2 phosphorylation. This study depicted that TGF-β1–induced collagen synthesis is dependent on TAK1 and its inhibition by A20 leads to the suppression of fibrosis and collagen synthesis. Another consequence of hypertrophy is myocyte apoptosis, it has been observed that A20 overexpression reduces the apoptosis and it is linked to cleavages of caspase 3, 9 and PARP as suggested by the previous study of its role in apoptosis (Zhou et al., 2017). This study postulates A20 as a good therapeutic target for cardiac hypertrophy.

## Potential Therapeutic UPS Interventions in Cardiac Hypertrophy

Despite dysregulation of UPS function in many diseases is much studied (Nalepa and Harper, 2003; Reinstein and Ciechanover, 2006; Wang and Maldonado, 2006; Bedford et al., 2008; Paul, 2008; Popovic et al., 2014) the involvement of UPS components in cardiac pathologies has been studied comparatively recently. Several proteins that are involved in a plethora of critical cellular pathways and play a vital function in the development of hypertrophic response in the heart are either component of the UPS or targeted by the UPS proteins (Nastasi et al., 2004; Schlossarek et al., 2014a). Proteasomal impairment could result in enhanced cellular levels of pro-hypertrophic and pro-apoptotic proteins. UPS dysregulation has been shown to activate the calcineurin-NFAT pathway and promoted maladaptive remodeling in cardiomyocytes (Tang M. et al., 2010). Furthermore, pro-apoptotic p53 levels were seen increased in human HCM and failing hearts compared with control heart in the context of impaired proteasomal activities (Sano et al., 2007; Birks et al., 2008; Predmore et al., 2010). Inhibitions well as activation of the UPS have been observed during cardiac hypertrophy in animal models (Depre et al., 2006; Tsukamoto et al., 2006; Meiners et al., 2008; Stansfield et al., 2008; Schlossarek et al., 2012, 2014a,b).

Components of the UPS or signaling pathways that involves the proteins of UPS have been targeted to develop novel therapies against different diseases (Ciechanover, 2006; Hoeller et al., 2006; Reinstein and Ciechanover, 2006; Paul, 2008; Bedford et al., 2011; Deshaies, 2014; Huang and Dixit, 2016; Gupta et al., 2018). A series of reports on partial proteasomal inhibition using low inhibitor doses have shown to inhibit cell cycle and survival of several different cell types (Lüss et al., 2002; Stangl et al., 2002). Treating mouse models using epoxomicin, an irreversible proteasome inhibitor attenuated the development of cardiac hypertrophy after transverse aortic constriction (TAC)-induced short-term pressure overload (Depre et al., 2006; Hedhli et al., 2008; Hedhli and Depre, 2009), similarly, genetically modified homozygous *Mybpc3*-targeted knock-in (KI) mice showed slight improvement in cardiac function after epoxomicin treatment (Schlossarek et al., 2014b). Low doses of another proteasome inhibitor MG132 suppressed isoproterenol- induced hypertrophy evident by reduction in induction of various hypertrophic markers such as, BNP, β-MHC, and SM α-actin as well as suppression of key signaling molecules such as Akt, calcineurin and ERK1/2 (Meiners et al., 2008), which may the reason of the suppressed hypertrophy as demonstrated by many studies both *in vivo* and *in vitro* (Sugden, 1999; Shioi et al., 2000; Bueno et al., 2002; Maillet et al., 2013; Bian et al., 2014; Jiang et al., 2015; Lee et al., 2016). Chen et al. (2010) inferred using long-term treatment of MG132 that an alleviated cardiac hypertrophy in AAB rats is a result of activation of ERK1/2 and JNK1 signaling pathways without affecting blood pressure. In addition, partial inhibition of the proteasome with low and non-toxic doses of bortezomib (Velcade) effectively attenuated pressure-overload-induced hypertrophy in hypertensive Dahl-salt sensitive rat hearts both *in vivo* and *in vitro* (**Table 2**) (Meiners et al., 2008). Ang II-induced hypertensive models showed inhibited hypertrophic phenotype via stabilizing Ang II type 1 receptor-associated protein (ATRAP) and inactivation of the p38 MAPK and STAT3 signaling pathways when treated with bortezomib, however, of the study didn’t extend to *in vivo* analysis (Li N. et al., 2015). Likewise, proteasome inhibitors MG132 and bortezomib suppressed cardiac hypertrophy induced by cholesterol and decreased expression of hypertrophic marker genes by inhibiting ERK and Akt activation (Lee et al., 2016). Treatment with the irreversible proteasome inhibitor PS-519 significantly prevented the development and promoted its regression of isoprenaline-induced hypertrophy by blocking the IκB-degradation effectively preventing nuclear translocation and activation of NF-κB in mice (**Table 2**) (Stansfield et al., 2008). Similar *in vivo* inhibition of NF-κB by pyrrolidinedithiocarbamate (PDTC) attenuated cardiac hypertrophy (Li et al., 2004; Gupta and Sen, 2005; Gupta et al., 2005).

**Table 2:** List of Inhibitors/drugs known so far against cardiac hypertrophy.

Inhibitor/drug	Target function	Reference
Epoxomicin	Suppression of the pressure loaded cardiac hypertrophy by an irreversible proteasome inhibition	Depre et al., 2006; Hedhli et al., 2008; Hedhli and Depre, 2009; Schlossarek et al., 2014b
MG132	Suppression of cardiac hypertrophy in AAB rats as a result of the activation of ERK1/2 and JNK1 signaling pathways	Sugden, 1999; Shioi et al., 2000; Bueno et al., 2002; Meiners et al., 2008; Maillet et al., 2013; Bian et al., 2014; Jiang et al., 2015; Lee et al., 2016
MG132	Suppression of cardiac hypertrophy in AAB rats is a result of activation of ERK1/2 and JNK1 signaling pathways without affecting blood pressure	Chen et al., 2010
MG132	Suppression of cholesterol induced cardiac hypertrophy by inhibition of ERK and Akt	Lee et al., 2016
Bortezomib	Suppression pressure-overload-induced hypertrophy in hypertensive Dahl-salt sensitive rat hearts both *in vivo* and *in vitro*	Meiners et al., 2008
Bortezomib	Inhibition of hypertrophic phenotype via stabilizing Ang II type 1 receptor-associated protein (ATRAP) and inactivation of the p38 MAPK and STAT3 signaling pathways	Li N. et al., 2015
Bortezomib	Suppression of cholesterol induced cardiac hypertrophy by inhibition of ERK and Akt	Lee et al., 2016
PS-519	Regression of isoprenaline-induced hypertrophy by the activation of NF-κB	Stansfield et al., 2008
Pyrrolidinedithiocarbamate (PDTC)	Suppression of pressure-overload-induced hypertrophy by inhibition of the NF-κB signaling pathway	Li et al., 2004; Gupta and Sen, 2005; Gupta et al., 2005
Auranofin	Attenuation of development of hypertrophy by the inactivation of the NF-κB pathway through the inhibition of IκBα protein degradation	Hu et al., 2018
Rapamycin	Attenuation of cardiac hypertrophy by promoting myocardial autophagy in a MEK/ERK signaling pathway dependent manner	Gu et al., 2016

It is clear from the above mentioned as well as several other studies that NF-κB plays a vital role in cardiac hypertrophy and heart failure, however, future studies will provide more evidence of its exact role and developing it as a potential therapeutic target in these diseases. Several compounds have been reported to block NF-κB signaling though without specificity. Blocking NF-κB signaling may prove to be of benefit in some cardiomyopathies, maintaining the NF-κB activity is also paramount in immune and inflammatory responses and homeostasis. Therefore, attempts to inhibit NF-κB signaling for therapeutic purposes in humans should be handled with great care.

Since UPS components are involved in a plethora of important cellular processes, targeting this system for therapy is not straightforward. Conflicting reports are documented regarding the role of proteasome inhibition in preventing cardiac hypertrophy. Chronic proteasome inhibition by a cell-permeable proteasome inhibitor, MG-262 activated the calcineurin–NFAT (Nuclear factor of activated T cells) pathway and induced LVH in sham-operated controls but hypertrophy and heart failure at high doses resulting in premature death in mice after TAC treatment (Tang M. et al., 2010). Similarly, administration of reversible inhibitor MLN-273 (Millennium Pharmaceuticals, Cambridge, MA, United States), an analog of bortezomib, led to LVH, diastolic dysfunction and a reduction in cardiac output in pigs along with an enhancement in cardiac apoptosis and fibrosis (Herrmann et al., 2013). Importantly, there also have been growing evidence of increase in the occurrence of cardiac complications, ranging from the inception of arrhythmias to the heart failure in aged individuals or patients with preexisting cardiac problems when treated with bortezomib (Richardson et al., 2005; Voortman and Giaccone, 2006; Berenson et al., 2007; Orciuolo et al., 2007; Hacihanefioglu et al., 2008; Bockorny et al., 2012). Even the second generation of proteasome inhibitors (e.g., carfilzomib) has also been reported to exhibit clinical cardiotoxicity (Grandin et al., 2015). However, some of the proteasome inhibitors (such as MG132) could be employed as components of anti-hypertensive drugs, exclusively opposing to LVH, without affecting the high blood pressure levels, reducing the major risk factors induced by systemic hypertension (Chen et al., 2010).

An alternative approach would be to target E3 ubiquitin ligases and DUBs and their particular signaling pathways to stimulate or block UPS-mediated protein degradation that may prove to be beneficial in targeting various cardiac afflictions. For example, blocking ubiquitination and subsequent proteasomal degradation of cyclin-dependent kinase inhibitor p27 by inhibiting its E3 ligase SCF-SKP2 using a specific inhibitor could result in inhibiting its effect on inducing pathological cardiac hypertrophy (Hauck et al., 2008; Wu et al., 2012).

Also, TRAF6 may contemplate as a potential therapeutic target owing to its crucial involvement in cardiac hypertrophy. There is available literature to demonstrate TRAF6 targeting in cardiomyopathies such as targeted blockage of CD40 and TRAF6 interaction used in the case of atherosclerosis in mouse models (Lutgens et al., 2010). At least 7 inhibitors were identified to block CD40-TRAF6 interaction, that reduces NF-κB signaling and lower IL-6 and IL-1β levels in RAW cells and in CD40-stimulated bone marrow-derived macrophages, respectively (Zarzycka et al., 2015; Abdullah et al., 2017). In addition, inhibition of TAK1 binding to TRAF6 has been shown to inhibit TAK1 mediated JNK1/2 and p38 signaling and subsequently inhibiting cardiac hypertrophy both *in vivo* and *in vitro (*Ji et al., 2016). Thus, targeting TRAF6 either directly or approaches to disrupt its binding with TAK1 may also hold promise in treating pathological hypertrophy of the heart. However, given the importance of TRAF6 throughout the body, cardiac-specific inhibition would likely be necessary.

In contrast to other deubiquitinating enzymes, USP14 has demonstrated to induce cardiac hypertrophy via enhancing GSK-3β phosphorylation, suggesting that USP14 can be potentially developed as a therapeutic target against cardiac hypertrophy (Liu et al., 2016). Treatment with a 19S proteasome-associated deubiquitinase inhibitor, Auranofin, which is a well-known drug against rheumatic arthritis has shown to attenuate the development of left ventricular hypertrophy both *in vitro* and *in vivo* (Hu et al., 2018). The antihypertrophic effect of the Auranofin might be attributed to the inhibition of IκBα protein degradation, which subsequently leads to the inactivation of the NF-κB pathway (Hu et al., 2018). However, so far, no molecule specifically targeting a cardiac E3 ubiquitin ligase or a DUB has been entered in clinical trials.

As myocardial autophagy contributes vitally to the cardiac hypertrophic response, therapeutic approaches targeting autophagy can prove to be effective against the disease. Rapamycin, an antifungal agent, and immunosuppressant drug, also an inhibitor of mTOR has shown to protect against cardiomyocyte hypertrophy both *in vitro* and *in vivo* by promoting myocardial autophagy through a mechanism involving the up-regulation of Noxa and Beclin-1 expression in a MEK/ERK signaling pathway dependent manner (**Table 2**) (Gu et al., 2016). However, a contrasting role of autophagy in elevating the cardiac disease is also known (Li Z. et al., 2015). Therefore, the therapeutic aspect of autophagy players in cardiac hypertrophy required detailed investigation.

## Discussion and Future Prospective

Post-translation modifications modulate the function of many proteins in the cell by regulating different signal transduction pathways. Ubiquitination is one of the post translation modification affecting various cellular pathways by tagging substrate protein with different ubiquitin chain attachments. Ubiquitination modification is known to work in coordination with other post translation modifications such as acetylation and phosphorylation in several cases to regulate signaling pathways. The function of ubiquitination in degradation pathway and other multiple cellular roles directed the role of this machinery in the heart. For maintaining the homeostatic protein level of cardiac structure protein degradation plays a vital role and additional role in other physiological functions of the heart. Cardiac hypertrophy is one of the leading causes of the cardiovascular disease that progresses into heart failure and subsequent death if left untreated. Although much is known about the pathways that regulate hypertrophic responses, the regulatory proteins that can be targeted to avert the development of heart failure resulting from cardiac hypertrophy are poorly defined. There is growing literature highlighting the role of UPS in heart failure and cardiomyocyte dysfunction. Identification of components of UPS particularly, E3 ligases and DUBs in the heart has enlightened their role in cardiac hypertrophy. There is a need to identify UPS players and to understand that how protein balance in the heart is achieved by E3 ligases and DUBs and their imbalance might result in the hypertrophy. Recent evidence traces the link between the UPS and cardiac hypertrophy and targeting the specific E3 ligases or DUBs by designing inhibitors against them, considering this would be a novel approach to treat cardiac diseases. Functions of various E3 ligases such as MDM2, Atrogin-I, TRIM32, MuRF-I, TRAF2, TRAF3, TRAF5 and TRAF6 and DUBs such as USP4, USP18, USP14, CLYD, and A20 have been proven to either exaggerate or attenuate cardiac hypertrophy phenotype in response to the overload situation. These documented E3 ligases and DUBs regulates various signaling pathways that are known to participate in the development of cardiac hypertrophy. Still, out of 700 E3 ligases and 100 DUBs expression profile of many of them and their role in normal heart function or in diseases state is not fully characterized. Future study in checking the level of these proteins in normal and disease stage such as hypertrophy of the heart would really pave a new way of understanding the disease with novel perspective. In many of the diseases such as cancer the E3 ligases and DUBs are explored and are suggestive to be used as a biomarker, similarly, their role can be studied in cardiac hypertrophy for biomarker application. Many of the available studies have highlighted the controversial role of the proteasome inhibitors in the reversal of cardiac hypertrophy. While such approaches are not cardiac specific and have negative effects associated with them. Alternatively targeting specific E3 ligases or DUBs will provide a novel strategy for the therapeutic interventions to treat the disease. However, more experimental investigations are needed to identify the substrates of the E3 ligases and DUBs responsible for cardiac hypertrophy, which would help in regulating their level of degradation as well as in designing inhibitors blocking E3 ligases or DUB-substrate interaction. Further, identification of the UPS components in the cardiac disease would provide more insight into the signaling pathway and also improve the treatment methods available to treat cardiac diseases.

## Author Contributions

IG and NV performed the bibliographic search. NV designed the figures. IG made the tables. SK designed and supervised all the work. All authors wrote, read, and approved the manuscript.

## Conflict of Interest Statement

The authors declare that the research was conducted in the absence of any commercial or financial relationships that could be construed as a potential conflict of interest.
