# Corrigendum: Emergence of Members of TRAF and DUB of Ubiquitin Proteasome System in the Regulation of Hypertrophic Cardiomyopathy

**DOI:** 10.3389/fgene.2018.00409

**Published:** 2018-09-19

**Authors:** Ishita Gupta, Nishant K. Varshney, Sameena Khan

**Affiliations:** ^1^Structural Immunology Group, International Centre for Genetic Engineering and Biotechnology, New Delhi, India; ^2^Drug Discovery Research Center, Translational Health Science and Technology Institute, Faridabad, India

**Keywords:** hypertrophy, E3 ligases, TRAFs, DUBs, ubiquitination, ubiquitin proteasome system

In the Author Contributions statement, the individual contribution of SK was inadvertently missed. SK designed and supervised all the work.

The authors apologize for this error and state that this does not change the scientific conclusions of the article in any way.

The original article has been updated.

## Conflict of interest statement

The authors declare that the research was conducted in the absence of any commercial or financial relationships that could be construed as a potential conflict of interest.

